# Examining the role of civic attitudes in the link between family wealth and school dropout among tertiary vocational students

**DOI:** 10.1038/s41539-023-00189-4

**Published:** 2023-09-15

**Authors:** Catrin Finkenauer, Maartje Boer, Jenna Spitzer, Dominic Weinberg, Kirsten Visser, Merel Jonker, Gonneke W. J. M. Stevens

**Affiliations:** 1 Department of Interdisciplinary Social Science, Utrecht University, Padualaan 14, 3584 CH Utrecht, The Netherlands; 2 Department of Psychology, Utrecht University, Utrecht, The Netherlands; 3 Clinical Child and Family Studies, Erasmus School of Social and Behavioural Sciences, Erasmus University Rotterdam, Rotterdam, The Netherlands; 4 Department of Human Geography and Spatial Planning, Faculty of Geosciences, Utrecht University, Utrecht, The Netherlands; 5 Department of Law, Faculty of Law, Economics and Governance, Utrecht University, Utrecht, The Netherlands

**Keywords:** Education, Social sciences

## Abstract

This study examined the relationship between family wealth and school dropout among vocational education students (*n* = 1,231; mean age=17.81). It investigated whether (1) family affluence and adolescents’ own perceptions and experiences of their family wealth (i.e., perceived family wealth, financial scarcity) predict dropout, (2) adolescents’ civic attitudes (i.e., system justification, institutional trust) explain the association between family wealth and school dropout, and (3) trust in teachers buffers against the risk of dropout among students with lower civic attitudes. Multivariate models revealed that financial scarcity predicted dropout. Financial scarcity showed an indirect only effect on dropout through lower institutional trust, but not through system justification. Trust in teachers was neither associated with dropout, nor a moderator. Controlling for mental health problems did not affect these results. This study helps explain how students’ experienced and perceived family wealth can affect their educational attainment, by reducing their trust in social institutions.

## Introduction

The importance of combating school dropout can hardly be overestimated. Especially in vocational education, school dropout rates are high in most countries^1^. Given the value of educational degrees and diplomas in current societies, adolescents who drop out of school are at risk for significant disadvantage throughout their adult life. Research spanning the social sciences shows that dropout predicts worse economic prospects for the future, which coincide with unemployment, financial hardship, reduced access to material and social resources, unhealthy lifestyles, poorer wellbeing, and worse health^2–4^. Therefore, the consequences of school dropout can be far-reaching and damaging for the individual student. Moreover, dropout has considerable societal costs, as the loss of talent and human capital forecasts a large societal economic burden throughout the life course^5,6^. Advancing our understanding of the processes underlying school dropout is therefore crucial. It can reveal leverage points for effective approaches to developmentally informed interventions to promote retention and academic achievement^7^. It can also reveal leverage points for breaking mechanisms that perpetuate social and educational inequalities.

The link between socioeconomic status (SES) and school dropout has been widely demonstrated in research. Studies consistently show that adolescents from lower SES backgrounds are more likely to drop out of school than their counterparts from higher SES backgrounds^8^. SES encompasses various aspects of economic and social resources, such as parental education, occupation, and income. Adolescents are sometimes unwilling or unable to reveal information about parental SES^9^, for example, because they have limited knowledge or understanding of their parents’ occupation or education^10^. Therefore, we conceptualize SES as family wealth, often operationalized as family affluence, which can be assessed by concrete questions adolescents know the answer to (e.g., having a dishwasher at home). Family affluence is a valid indicator of economic resources and assets available to a family^11^ assessing SES among adolescents^12^. Additionally, recent research shows that family wealth is an important determinant of educational attainment, above and beyond parental education^13–15^.

While a combination of factors is likely at play^6,8^, the role of adolescents’ civic attitudes in explaining the link between family wealth and school dropout has not yet received much attention. Civic attitudes concern young people’s feelings regarding their roles and position in society, such as their belief that society is just, trust in institutions, and understanding of socioeconomic inequalities^16–18^. Adolescents’ civic attitudes, in turn, can shape their academic motivation and educational behavior^19,20^. Extending these suggestions, we expect that civic attitudes can influence whether adolescents drop out of school.

In this study, we focus on dropout among students in vocational education, evaluating whether family wealth is associated with adolescents’ civic attitudes, and whether these attitudes, in turn, impact dropout. Specifically, we predict that adolescents with lower family wealth (i.e., lower family affluence, perceived family wealth and financial scarcity) report lower system justifying beliefs and institutional trust, and that lower levels of these civic attitudes increase their risk of school dropout. To inform potential interventions, we explore whether higher trust in teachers can be a protective factor for adolescents against the risks associated with lower civic attitudes for school dropout.

Adolescents with lower *family wealth*—lower family income, affluence, and fewer material possessions^13^—are at significant higher risk of dropping out of school than students with more family wealth^8,21^. To illustrate, in a prospective Danish cohort study, adolescents from families with the lowest socioeconomic position (a composite measure of highest attained education and household income) had approximately a 3-fold higher risk of not completing a secondary education compared to adolescents from the highest socioeconomic position^22^. Empirical research examining the association between family wealth and educational attainment and dropout underlines the persistence of intergenerational inequalities and shows that family SES and wealth have direct and indirect associations with adolescents’ educational attainment and dropout^23–26^.

Research has made great inroads in explaining the link between family SES and school dropout by focusing on more objective indicators of family wealth, such as family income or affluence. However, it has neglected the unique characteristics of the developmental period of adolescence. During adolescence, young people become increasingly aware of their (family’s) socioeconomic position in society and compare it with that of others. They also begin to shape their own socioeconomic position^17,27^. Therefore, considering adolescents’ own perceptions and experiences of their (family’s) socioeconomic positions is crucial. *Perceived family wealth* represents adolescents’ subjective perception of how well-off their family is compared to others. Perceived family wealth often emerges as a stronger predictor of adolescent behavior and wellbeing than more objective indicators of family SES^28,29^. *Financial scarcity* concerns adolescents’ subjective experience of having limited financial resources or experiencing financial instability required to cope with personal demands and needs^30^. Financial scarcity can be stressful and is associated with a range of negative psychological and behavioral consequences^31^, especially among younger people^32^, which may also contribute to dropout.

Studies that attempt to explain the negative association between SES and dropout in vocational education yield multiple factors that play a role in increasing the risk of lower educational attainment and dropout, including adolescents’ lower cognitive abilities, less access to material, social, and cultural resources, behavioral problems, and lower educational expectations^23–26,33^. However, civic attitudes may play an important mediating role in this association as well. As adolescents engage with institutions, such as schools and community organizations, they learn and practice the norms of the societies they live in^16,34,35^. Positive and negative interactions with institutions transmit norms of (the lack of) fairness, social responsibility, and social justice, which adolescents internalize to develop their civic attitudes^16^ and critical consciousness (i.e., awareness and understanding of the social, political, and economic forces that contribute to inequality, oppression, and injustice in society^27,36^). Compared to adolescents with higher family wealth, adolescents with lower family wealth are more likely to face more stressful and threatening family, school, and neighborhood environments. Their families may also have less access to supportive jobs and health services and face more discriminatory policing and legal systems^37^. Such structural and experienced disadvantages may lead adolescents from lower SES backgrounds (as measured by parental education) to perceive society as more unequal^38^. Greater family wealth is associated with higher beliefs in the fairness and legitimacy of a country’s institutions among adolescents^39,40^. Having a positive view of societal fairness and endorsing *system-justifying beliefs* may lead young people to believe that they have control over their own outcomes^41,42^, which may motivate students to work harder^43^ and achieve better grades^44,45^.

Economic and social uncertainty, including income inequality, job insecurity, and lack of economic growth, is a strong predictor of low institutional trust, especially among younger people^46^. Not surprisingly, lower family wealth, economic uncertainties associated with lower SES, and biased policy-responsiveness towards higher SES^47^ negatively shape adolescents’ trust in institutions (e.g., government, police) and weaken their belief in, and motivation to achieve, socioeconomic success^19,48,49^. Lower institutional trust may therefore reduce the likelihood that adolescents engage in behaviors associated with success, including staying in school^21^, and persisting in the face of academic setbacks. Thereby, lower institutional trust associated with lower family wealth in adolescence may increase the risk for school dropout.

Trusting relationships with caring, reliable adults play an important role in promoting adolescent wellbeing, reducing risk behaviors, regulating their emotions, and focusing on their future especially among adolescents with lower SES^50,51^. In particular, relationships with teachers whom adolescents trust are important for their behavioral engagement at school and school outcomes^50–53^. Students who trust and feel emotionally supported by their teachers are more engaged in school, have higher grades and attendance, and are less likely to dropout^54^, especially among adolescents who are at high risk of experiencing difficulties^44^.

In addition, teachers may be able to mitigate the negative impact of lower civic attitudes on school dropout by promoting fairness and justice in the classroom. Teachers can be seen as the most important representatives of social institutions for young people. A trusted teacher can demonstrate that there are professionals who are reliable and fair and who apply transparent rules, which can buffer the effect of adolescents’ perception that systems are unfair and/or untrustworthy^55^. Moreover, trusted teachers may reduce the risk of dropping out among students with lower civic attitudes by creating a protective and supportive classroom environment^56^. Teachers can promote open and respectful dialogue and model critical consciousness by engaging in critical reflection themselves. In such a protective environment, students with lower civic attitudes may come to experience rules as fair and legitimate. Therefore, higher interpersonal trust in teachers may be an important protective factor that reduces the likelihood of dropout when adolescents believe that systems are unfair or untrustworthy.

Research has shown that *mental health problems* (i.e., emotional symptoms, conduct problems, hyperactivity/inattention, and peer problems) are related to SES^26^ and school dropout^8^. Additionally, research suggests that *migration background* may be associated with family SES^57,58^ and relate to dropout from vocational education^34^. To examine whether our expectations hold above and beyond these factors, we will control for mental health problems and migration background.

This pre-registered study (osf.io/ezjuf) examines associations between family wealth, civic attitudes, and school dropout in a large sample of vocational students in the Netherlands. We extend existing research on the association between SES and school dropout in three important ways. Firstly, recognizing the crucial importance of adolescence as a developmental period in which adolescents come to understand their social position, we distinguish between family wealth and adolescents’ own subjective perceptions of family wealth and own experienced financial scarcity. Secondly, and related to adolescents’ increasing societal awareness, we examine the role of civic attitudes (i.e., system justification beliefs and lower trust in institutions) in the association between family wealth and school dropout. Thirdly, we examine whether interpersonal trust in teachers – a potential target for intervention – can buffer against the expected increased likelihood of school dropout when adolescents have lower civic attitudes. Fourthly, we examine our hypotheses in vocational schools where dropout is particularly high.

## Results

### Descriptive Analyses

Table 1 shows the descriptive statistics for all study variables. The sample consisted of 1,231 adolescents (*M*_age_ = 17.81, *SD*_age_ = 1.82, 44.31% male, 74.53% without a migration background) of whom 10.39% dropped out of education after their first year. For financial scarcity, mean scores were below the midpoint of the scale; mean scores for perceived family wealth, institutional trust, and system justification were around the midpoint, and mean scores were above the midpoint for trust in teachers. In Table 2, correlations between all study variables are presented. Lower levels of family wealth, system justification, institutional trust, and trust in teachers were associated with more school dropout, while the same was true for higher levels of financial scarcity, emotional problems, conduct problems and hyperactivity/inattention. All these effect sizes were small. System justification and institutional trust were moderately strongly positively correlated (*r* = 0.53). Peer problems, age, gender, and migration background were not significantly associated with dropout, and therefore not included as covariates in our models.

**Table 1:** Descriptive statistics.

Variable	Obs.	% missing	Mean/%	*SD*	Min.	Max.	α	ICC
School dropout	1059	13.97	10.39%		0	1		0.10
Family affluence	1206	2.03	0.50	0.28	0.00	1.00	0.72	0.08
Perceived family wealth	1199	2.60	3.02	0.74	1	5		0.03
Financial scarcity	1206	2.03	2.25	0.79	1	5	0.78	0.06
System justification	1144	7.07	4.34	0.99	1	7	0.90	0.03
Institutional trust	1167	5.20	5.36	1.91	0	10	0.89	0.03
Trust in teachers	1167	5.20	3.64	0.73	1	5	0.83	0.09
Emotional problems	1192	3.17	3.08	2.53	0	10	0.82	0.10
Conduct problems	1191	3.25	0.94	1.35	0	10	0.58	0.00
Hyperactivity/ inattention	1193	3.09	4.46	3.02	0	10	0.79	0.03
Peer problems	1192	3.17	2.61	1.94	0	10	0.53	0.01
Age	1227	0.32	17.81	1.82	16.00	29.83		0.20
Male	1230	0.08	44.31%		0	1		0.54
Migration background	1225	0.49	25.47%		0	1		0.18

*Obs.* observations, *SD* standard deviation, *Min.* minimum, *Max.* maximum., α alpha (internal consistency), *ICC* intraclass correlation school class level (*n* clusters = 71).

**Table 2:** Pairwise correlations.

		1 School dropout	2 Family affluence	3 Perceived family wealth	4 Financial scarcity	5 System justification	6 Institutional trust	7 Trust in teachers	8 Emotional problems	9 Conduct problems	10 Hyper-activity/ inattention	11 Peer problems
**1**	**School dropout**	1.00										
**2**	**Family affluence**	−0.13*	1.00									
**3**	**Perceived family wealth**	−0.09	0.41***	1.00								
**4**	**Financial scarcity**	0.14**	−0.21***	−0.41***	1.00							
**5**	**System justification**	−0.14**	0.09**	0.22***	−0.28***	1.00						
**6**	**Institutional trust**	−0.21***	0.13***	0.20***	−0.23***	0.53***	1.00					
**7**	**Trust in teachers**	−0.11*	−0.02	0.06*	−0.09**	0.29***	0.36***	1.00				
**8**	**Emotional problems**	0.15**	−0.10**	−0.16***	0.37***	−0.25***	−0.21***	−0.16***	1.00			
**9**	**Conduct problems**	0.15**	−0.01	−0.04	0.22***	−0.20***	−0.22***	−0.18***	0.25***	1.00		
**10**	**Hyperactivity/ inattention**	0.15**	0.09**	0.01	0.17***	−0.14***	−0.12***	−0.12***	0.32***	0.28***	1.00	
**11**	**Peer problems**	0.08	−0.05	−0.11***	0.20***	−0.22***	−0.23***	−0.11***	0.36***	0.21***	0.12***	1.00
**12**	**Age**	0.09	−0.14***	−0.18***	0.21***	−0.04	−0.11***	0.06*	0.05	0.04	−0.07*	0.14***
**13**	**Male**	0.10	0.09*	0.10**	−0.05	0.01	−0.04	0.09*	−0.37***	0.12**	−0.03	−0.04
**14**	**Migration**	0.08	−0.29***	−0.28***	0.00	0.04	−0.16***	0.03	−0.15**	−0.02	−0.18***	0.00

* *p* < 0.05, ** *p* < 0.01, *** *p* < 0.001

### Family Wealth and School Dropout

In our univariate models (M1a-c), lower levels of family affluence and higher levels of financial scarcity increased adolescents’ probability of dropping out of school, but lower levels of perceived family wealth did not (Table 3). The association between family affluence and dropout disappeared when controlling for financial scarcity (M1d).

**Table 3:** Results logistic regression, school dropout.

			School dropout			
Model	*n*^a^	Family wealth indicator	*B*	*SE*	*p*	*OR*
1a	1051	Family affluence	**−0.890**	**0.399**	**0.026**	**0.411**
1b	1045	Perceived family wealth	−0.250	0.158	0.113	0.779
1c	1051	Financial scarcity	**0.358**	**0.147**	**0.015**	**1.430**
1d^b^	1216	Family affluence	−0.705	0.389	0.070	0.494
		Perceived family wealth	0.001	0.156	0.996	1.001
		Financial scarcity	**0.303**	**0.135**	**0.025**	**1.354**

*SE* standard error; *p* p-value; *OR* odds ratio. Boldface rows denote significant associations.

^a^Because full information maximum likelihood was used, sample size *n* varied depending on the included variables.

^b^The model included covariances between the three Family wealth indicators (not shown in Table).

### Mediation by Civic Attitudes

**Fig. 1:**
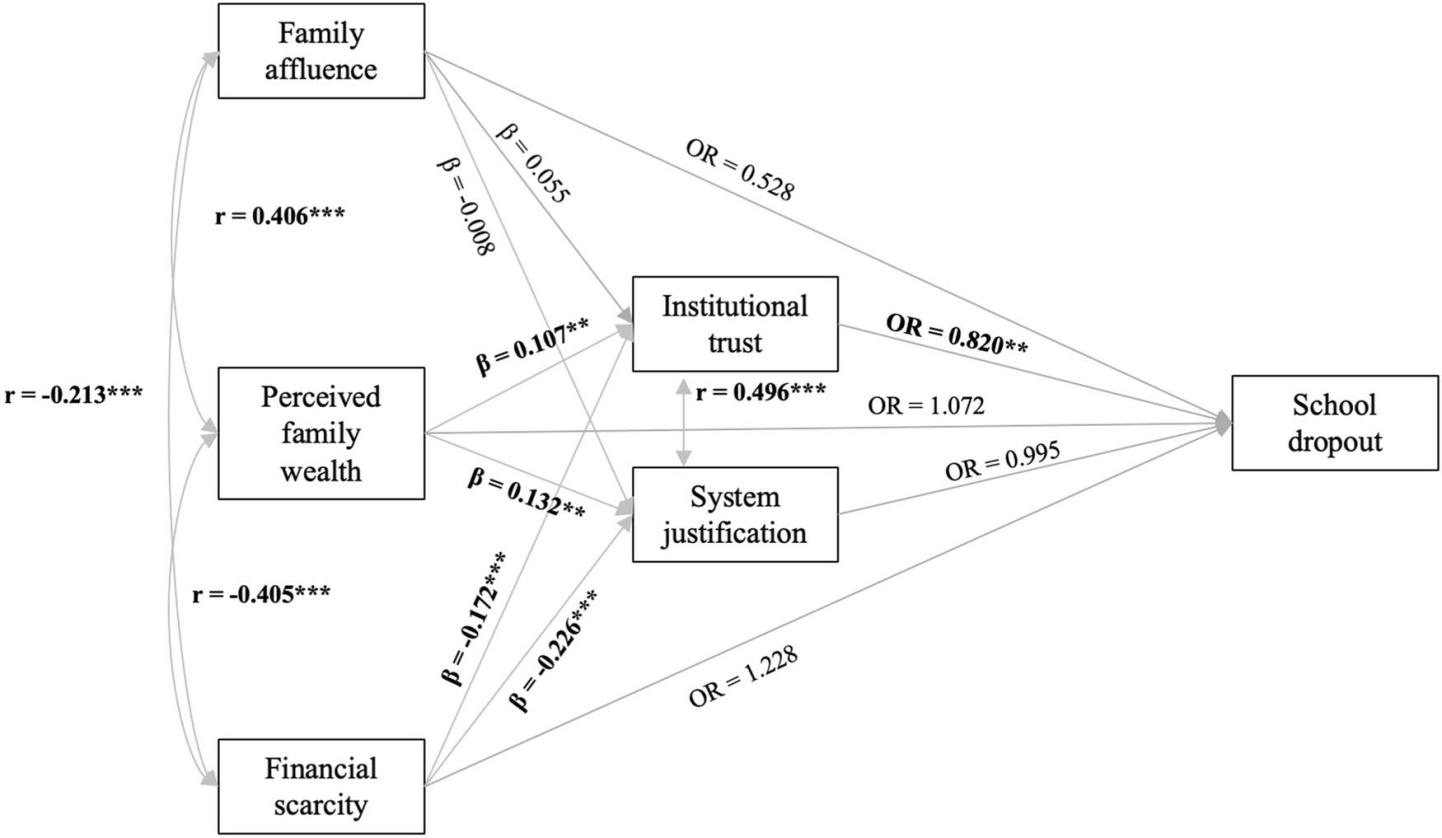
Path model results of Model 2c (n = 1,218). Diagram shows odds ratios (*OR*), standardized coefficients (*β*), and correlations (*r*). Two indirect effects were found (1) effect of perceived family wealth on dropout via institutional trust: OR = 0.947, LL = 0.893, UL = 0.988, (2) effect of financial scarcity on dropout via institutional trust: OR = 1.086, LL = 1.025, UL = 1.169.

Compared to the model including all three indicators of family wealth (M1d), the mediation models (M2a-c) showed significant model fit improvement, with the strongest improvement for Model 2c, which included both mediators simultaneously (see Supplementary Table 1). Hence, model estimates from model M2c were used for interpretation and are displayed in Fig. 1.

**Fig. 2:**
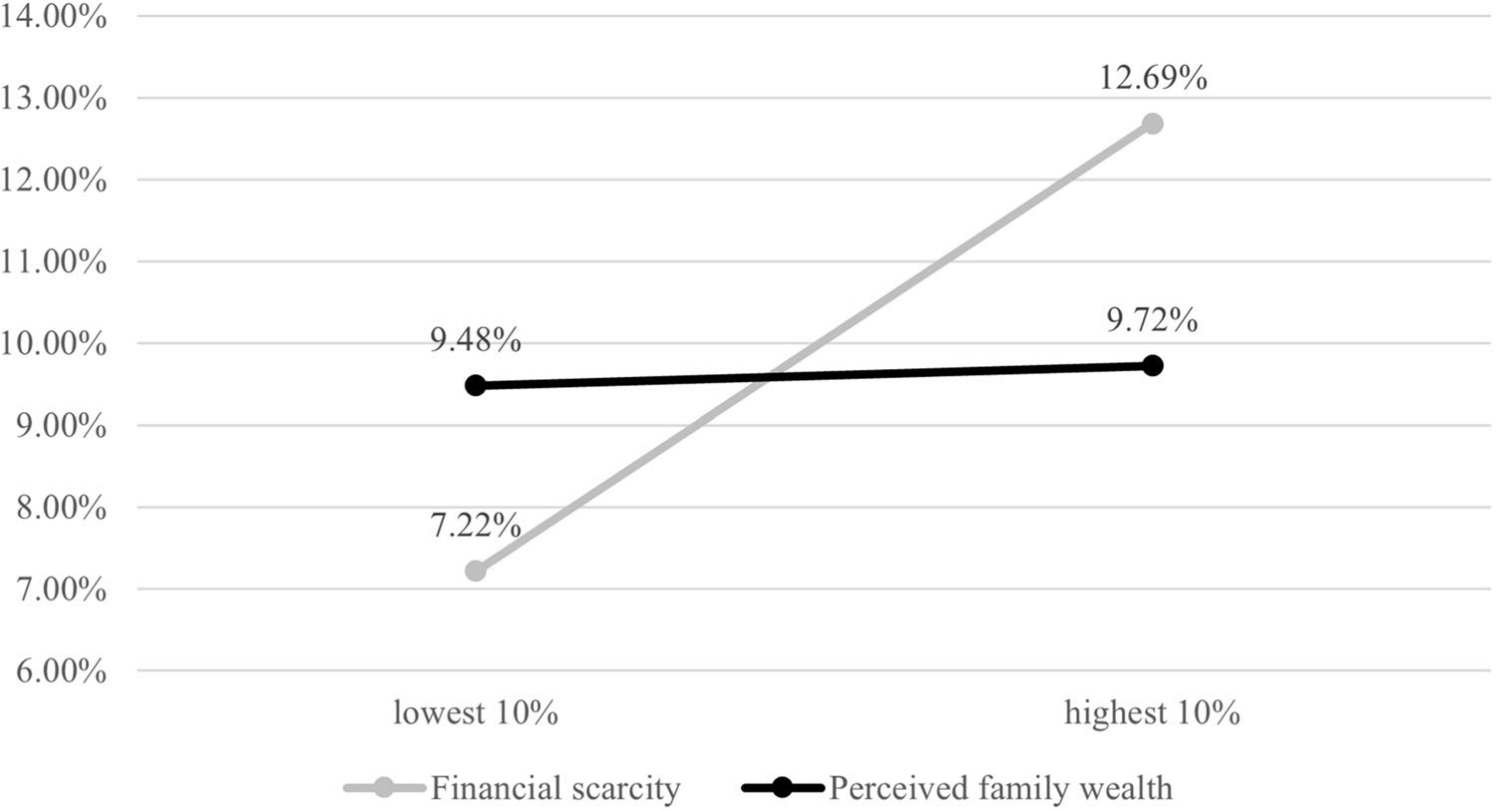
Probability of school dropout, by family wealth indicators (n = 1,218). Predicted probabilities were based on the total effects from Model 2c, calculated while holding all covariates at their means.

In the mediation model M2c, the previously found direct effect of financial scarcity on dropout (M1d) disappeared. However, an *indirect only mediation* was observed (Zhao, 2010), whereby higher financial scarcity was associated with lower levels of institutional trust, which, in turn, predicted a higher probability of dropout (M2c: *B*_indirect_ = 0.083, 95% CI [0.024, 0.156], OR = 1.086). The total effect of financial scarcity on dropout was also positive (M2c: *B*_total financial scarcity_ = 0.289, 95% CI [0.034, 0.555], OR = 1.335), indicating a significantly greater risk for dropout when adolescents experienced more financial scarcity. Illustrating this total effect, Fig. 2 shows that the probability of dropout is more than 1.5 times higher for adolescents with very high levels of financial scarcity (i.e., at the 90^th^ percentile), compared to adolescents with very low levels of financial scarcity (i.e., at the 10^th^ percentile): 12.69 versus 7.22%.

Although initial models did not show a direct effect of perceived family wealth on dropout (M1a-d), the mediation model M2c yielded an indirect only mediation whereby higher perceived family wealth decreased the probability of dropout through higher levels of institutional trust (M2c: *B*_indirect perceived family wealth_ = −0.055, 95% CI [−0.113, −0.012], OR = 0.947). Yet, the total effect of perceived family wealth was approximately zero (M2c: *B*_total perceived family wealth_ = 0.014, 95% CI [−0.218, 0.310], OR = 1.014; Fig. 2).

In Model 2b, both financial scarcity and low perceived family wealth were indirectly related to dropout through lower levels of system justification. However, these mediational paths attenuated to insignificance in Model 2c (i.e., once institutional trust was taken into account; Fig. 1).

### Moderation by Interpersonal Trust in Teachers

Adding trust in teachers as main effect and moderator of the effects of both indicators of civic attitudes on dropout did not improve model fit and their estimates were non-significant (M3; see Supplementary Tables 1 and 2). This means that the relation of institutional trust and system justification with dropout did not depend on trust in teachers.

### Confounding by Mental Health Problems

Adding conduct problems, emotional problems, and hyperactivity/inattention as additional covariates improved model fit (M4a-c; Supplementary Table 1), but the previously found indirect effects remained and the strength of the effects in M4a-c were comparable to those in M3. Furthermore, only hyperactivity/inattention predicted dropout significantly. Estimates from Models 2 until 4 can be found in Supplementary Table 2.

## Discussion

School dropout is one of the strongest predictors of life-course disadvantage, including lower earnings, poorer health, and more dependence on public assistance^6^. Past theory and research argued that family SES and the distinct ecologies associated with it are impactful predictors of dropout^19,20^. The results from this study dovetail with this prior work and expand upon it in important ways. Firstly, given the unique developmental phase of adolescence, it differentiated between family affluence and adolescents’ own perceptions and experiences of their and their family’s wealth. Secondly, it examined adolescents’ civic attitudes—beliefs about whether institutions are fair and can be trusted—as important but underexplored factor in explaining the pervasive association between family wealth and school dropout. Thirdly, it examined whether interpersonal trust in teachers can buffer against the increased risk of dropout among students with lower civic attitudes.

Replicating existing studies on socioeconomic inequalities in school dropout^8^, we found that family wealth predicted dropout over the course of the first year of tertiary vocational education. However, our results showed that when adding indicators reflecting adolescents’ own perceptions of their family wealth, the direct association between family affluence and dropout became non-significant. These findings suggest that adolescents’ own experiences of financial scarcity were a stronger driver of dropout than family affluence. Our second aim was to test whether civic attitudes (i.e., system justification beliefs and lower trust in institutions) mediated the association between family wealth and school dropout. We found that financial scarcity showed indirect only effects on dropout through lower levels of institutional trust, but not through system justifying beliefs. Additionally, although no direct nor total effects of perceived family wealth on school dropout were found, we did find an indirect effect whereby higher levels of perceived family wealth were associated with higher institutional trust, which subsequently predicted lower probability of school dropout. Our third aim was to explore the buffering potential of interpersonal trust in teachers. Though trust in teachers correlated negatively with dropout, in multivariate models it was neither associated with dropout, nor a moderator of the association between civic attitudes and dropout.

As indicated above, our findings reveal that, of a variety measures of family wealth, adolescents’ experienced financial scarcity is particularly predictive of school dropout. The fact that the association between family wealth and dropout disappeared when we controlled for financial scarcity suggests that the impact of family wealth on dropout is mediated by adolescents’ personal experience of scarce financial resources. Explanations for this finding may be found in research showing that the subjective perception of strained material and financial resources is especially associated with increased stress, worry, rumination, a heightened focus on short-term goals, and avoidant financial behaviors^28^, especially among younger people^32,59^. Moreover, dropping out of school to enter the job market may generate money, prestige, and economic stability^60^. In turn, stress, short-term goals, and the need for economic stability may have increased the likelihood of school dropout. Notably, the association between financial scarcity and school dropout did not disappear after adding different indicators of mental health to the model, indicating that other mechanisms may play a role. Additionally, people who perceive that they have less access to material resources feel that they have fewer choices and experience more external constraints placed on their decisions and behavior^61^. This perception of having less personal control may both weaken adolescents’ belief in and motivation to achieve academic success^19^. Furthermore, having a lower sense of personal control may, as we predicted, reduce adolescents’ civic attitudes, which ultimately may result in dropout.

We operationalized civic attitudes through system justifying beliefs and institutional trust; two related but distinct concepts. Their conceptual overlap is reflected in their high correlation (*r* = .53), as well as the finding that perceived family wealth and financial scarcity were negatively associated with both system justification and institutional trust. One key difference between these concepts is that institutional trust focuses on the trustworthiness of specific institutions, while system justification refers to beliefs about the overall fairness and legitimacy of the social, political, and economic systems in a specific country. This more abstract nature of system justifying beliefs, may explain why we did not find an association between system justifying beliefs and school dropout when controlling for trust in institutions.

It is also possible that institutional trust and system justification have differential effects on individuals or groups. To illustrate, across diverse groups and cultures, higher institutional trust is consistently associated with increased participation in political and civic activities, compliance with the law, and support of social norms^62^. System justification may not be beneficial for all adolescents, however. Because system justification buffers stress and serves to cope with unfair systems and institutions, higher system-justifying beliefs have been found to be associated with lower educational attainment among marginalized groups^27^. As compared to their better-off counterparts, when members of marginalized groups perceive systems and institutions as fair, this implies that they deserve their place in society, which may reduce their motivation and feelings of control, increasing their likelihood of school dropout.

Only institutional trust, but not system justification, mediated the association between financial scarcity and dropout. One explanation for this finding may be the impact of institutional trust on mental wellbeing^63^. Nevertheless, these associations did not disappear when controlling for mental health problems, suggesting that our found effects are robust and independent of adolescents’ mental health. As discussed above, diverse individual (e.g., lack of control, perceived constraints, short-term orientation), social (lower social support), material (e.g., private tutoring), and cultural (e.g., reading books) mechanisms may independently and jointly explain why lower SES is associated with higher school dropout. Our results suggest that some of these mechanisms are related to institutional trust. For example, adolescents with lower institutional trust may be more likely to believe that their school and the labor market are non-supportive, which can hinder a sense of belonging within the educational system and discourage academic engagement. Additionally, adolescents with lower institutional trust may be less likely to view their education as valuable and worth investing in, which can motivate them to dropout. The finding that institutional trust is a predictor of school dropout may encourage scientific and societal conversations not only about students’ institutional trust, but also about what institutions and their representatives can do to increase, and possibly repair, adolescents’ trust. Trust develops when (representatives of) institutions are trustworthy, when they show honesty, reliability, and competence^64^. Investigating the factors that facilitate (or undermine) trust may therefore enhance our understanding of structural conditions that contribute to wealth and educational inequalities^65^, informing policymakers and potential interventions to reduce dropout.

We found a small indirect negative effect of perceived family wealth on school dropout through institutional trust, but no total effect of perceived family wealth on dropout, which is not an uncommon finding for mediation analysis^66^. Conceptually, this finding suggests that there are multiple mediating factors at play in the association between perceived family wealth and school dropout, with some positive and some negative pathways.

We hypothesized that trusted teachers, who represent a crucial institution in society and constitute an important non-parent adult in the lives of adolescents, may be able to overcome the negative impact of civic attitudes on dropout. However, in our study, trust in teachers had neither a protective nor a promotive effect on dropout. Possibly, the role of teachers in tertiary vocational school in dropout is not as important as we expected. This may be the case because students in our sample had recently started their education or because the COVID-19 pandemic and associated regulations, which led to online education in the Netherlands for several months after the initial data collection in this study in March 2020, affected typical student-teacher relationships. Also, our measure assessed trust in teachers in general. Trust in specific teachers may be more important to adolescents’ behavioral engagement with school and school dropout^67^.

Furthermore, our findings revealed that experiencing financial scarcity was one of the most important drivers for dropout. The pandemic may have exacerbated financial scarcity. Especially for adolescents with very high levels of financial scarcity, dealing with the immediate financial challenges may have taken priority over the longer-term consequences of dropping out of school^30^ and thus teachers’ influence on students may have been relatively reduced. Or alternatively, other mechanisms may outweigh the importance of trust in teachers for school dropout, including poor social relations with friends and classmates, antisocial and/or delinquent behavior, lower parental involvement with school, lower parental expectations, and living in a non-intact household^8,22,23,68^.

The current study included a considerable sample of tertiary vocational students, who are at high risk of dropping out, and assessed objective dropout several months after assessing the predictors. Despite these strengths, the present study has limitations. First, the study was conducted in vocational schools in the Netherlands, and it is unclear whether the results generalize to other educational levels and countries. Second, although our findings may indicate that experiencing financial scarcity causes adolescents to develop lower institutional trust, and our hypotheses reflect such an assumption, they are inadequate to rule out alternative interpretations. Additionally, family wealth and civic attitudes were assessed at the same timepoint. Therefore, our findings are correlational and warrant replication with designs that allow researchers to tease apart their directionality. Third, our study assessed adolescent-reported family affluence using the FAS as an indicator of family wealth. Most recent studies have shown a high test-retest reliability and agreement between child and parent reports on the FAS-III items^12,13,69^ but have found moderate associations with family income. It is possible that family income is a slightly different concept than family affluence. It is also possible that adolescents’ understanding of family income and/or family affluence is limited. Indeed, researchers are increasingly recognizing the boundaries of adolescent proxy reports of family SES^70^, and research exploring other indicators of family wealth would be promising. Moreover, family wealth is one important aspect of family SES, but other aspects, including parental education, parental occupation, and their interaction need systematic attention in research concerning the role of civic attitudes in explaining the association between SES and school dropout^71^. Additionally, we assessed perceived family wealth with a single item, which may not capture the complexity of wealth perceptions. Fourth, our dropout measure did not differentiate between different types of dropouts, so we were unable to distinguish between students who dropped out of education entirely and those who switched to another type of training^72^. Perhaps our indirect effects were quite small because they conflate effects for both types of students. We expect that future research that includes only students who drop out of education entirely may find stronger effects. Finally, the findings for perceived family wealth, which was measured with a single item, and the (lack of) findings for the moderation of trust in teachers, which may require additional statistical power^73^, warrant replication.

Family wealth is a potent and pervasive predictor of dropout, shaping adolescents’ educational experiences. Our findings showed that rather than family affluence, adolescents’ experiences of financial scarcity predicted adolescents’ institutional trust. Institutional trust, in turn predicted dropout. Adolescents’ level of mental health problems did not affect this relation. Institutional trust is key for individuals and societies, and essential for almost all interactions between citizens and (representatives of) institutions. It has not yet received much attention in the literature on adolescent development and dropout. We hope that future research will build on our findings to further understand how socioeconomic disparities shape adolescents’ trust in institutions and their academic trajectories.

## Methods

### Sample and Procedures

We used data from the first wave of the ongoing longitudinal YOUth Got Talent project on the wellbeing of adolescents enrolled in the first year of tertiary vocational education in the Netherlands and combined these data with official dropout numbers after the first year. Tertiary vocational schools in the Netherlands are divided into four levels (1 – entry-level; 2 – basic, 3 – professional; 4 – middle-management). Pilot research revealed that adolescents in Level 1 classes were unable to complete the questionnaire satisfactorily, so they were not included in this study. Adolescents attended classes in three vocational schools and participated in training in fields such as creative, technical, and health education. Data were collected between September 2019 and February 2020. At T1, 1,519 students could have been included in the study, while 81% of them participated, yielding a sample of 1,231 adolescents (*M*_age_ = 17.81, *SD*_age_ = 1.82, 44.31% male, 74.53% without a migration background). Most of the non-participation was related to sickness/classroom absence (15%), and 4% of the non-participation was due to refusal or invalid responses.

Self-report questionnaires (96.5% digital; 3.5% paper-and-pencil) were administered in the classroom (*n* = 71 classes), taking roughly 20–30 minutes. Before data collection, participants gave active, written consent. In the Netherlands, adolescents aged 16 and over can consent to participate in research, and all participants were aged 16 and over. In October/November 2020, the three schools provided information to researchers on whether students had dropped out of school. This information was only available for students who had consented to this information being shared. Ethical approval was gained from the Ethics Assessment Committee of the Faculty of Social Sciences at Utrecht University (FETC18-070) in 2018.

### Measures

#### **School dropout**

*School dropout* was measured by the officially reported school registration data obtained in November 2020 (that is about 9 months to a year after the first data collection).

#### **Family affluence**

*Family affluence* reflects the objective material and financial resources in the family. We measured family affluence with the Family Affluence Scale (FAS^13^). The scale consists of six items about family material assets: car(s)/van(s), own bedroom, holiday(s) abroad, computer(s), dishwasher, and bathroom(s)^13^. For participants who completed all items, we summed item scores, then ridit-transformed the sum score into a continuous family affluence score (range = 0-1; mean = 0.5; ordinal *α* = 0.72)^74^, with a higher score indicating more material assets^75^. The FAS is a reliable and valid instrument for adolescents to report their family affluence^13^.

#### Perceived family wealth

Adolescents reported *perceived family wealth* by answering the question, “How well off do you think your family is?” on a 5-point response scale from 1 (*very well off*) to 5 (*not at all well off*). We reversed the scale so that higher scores indicated higher perceived family wealth. The measure is easy to answer for adolescents and reflects the subjective dimension of family SES^76^.

#### Financial scarcity

Adolescents’ experiences of financial scarcity were measured with 6 items of the Psychological Inventory of Financial Scarcity (PIFS)^59^. Adolescents indicated to what extent they agreed with each statement (e.g., “I often worry about money”) on a scale ranging from 1 (*completely disagree*) to 5 (*completely agree*). In our study, this measure showed good internal consistency, Cronbach’s *α* = .78. Higher mean scores indicated higher levels of financial scarcity.

#### System justification

Endorsement of system-justifying beliefs was measured using the system justification scale^27^ adapted to the Dutch context. Eleven items measured adolescents’ perceptions of fairness, legitimacy, and justifiability of the Dutch socio-political and economic system (e.g., “In general, Dutch society is fair”, “People get fair treatment in the Netherlands, no matter who they are”) with a 7-point Likert scale ranging from 1 (*totally disagree*) to 7 (*totally agree*). Higher mean scores indicated higher system justification beliefs (Cronbach’s *α* = 0.90).

#### Institutional trust

We measured adolescents’ trust in institutions using the 7-item institutional trust scale developed by the OECD^77^. Adolescents rated their trust towards institutions (i.e., “Dutch politicians”, “the police”, “health care professionals, such as doctors and psychologists”, “people who work for the government”, “the news”, “courts and judges”, and “information on social media”) on an 11-point scale, ranging from 0 (*not at all)* to 10 (*completely*). Higher mean scores indicated higher levels of institutional trust (Cronbach’s *α* = 0.89).

#### Interpersonal trust in teachers

We assessed interpersonal trust in teachers using the 3-item Teacher Support Scale^78^, which has good psychometric qualities^79^. On a 5-point scale ranging from 1 (*strongly agree*) to 5 (*strongly disagree*), adolescents rated the extent to which they trust their teachers (i.e., “I feel that my teachers accept me as I am”, “I feel a lot of trust in my teachers”, “I feel that my teachers care about me as a person”). Higher mean scores indicated higher levels of interpersonal trust in teachers (Cronbach’s *α* = .83).

#### Mental health problems

Adolescents reported *emotional symptoms*, *conduct problems*, *hyperactivity/inattention*, and *peer problems* on the SDQ-R: a revised version of the self-report Strengths and Difficulties Questionnaire (SDQ) that has better psychometric properties in adolescents than the original version^80,81^. The SDQ-R asks about behavior and feelings over the past six months – sample items are “I get very angry and often lose my temper” and “I worry a lot”. The SDQ-R has a 3-point ordinal response scale: 0 (*not true*), 1 (*somewhat true*), 2 (*certainly true*). It consists of 15 items measuring four subscales: emotional symptoms (5 items); conduct problems (4 items); hyperactivity/inattention (3 items); and peer relationship problems (3 items). In this study, two subscales, emotional symptoms (ordinal α = .82) and hyperactivity/inattention (ordinal α = 0.79), had good internal consistency^68^. Internal consistency for conduct problems (ordinal α = 0.58) and peer problems subscales (ordinal α = .53) was less adequate, in line with former research^57^. We computed mean scores, which were then multiplied by five to retain comparability with the original SDQ. Higher subscale scores indicated more problems (range 0 to 10).

#### Background characteristics

Adolescents reported whether they were a girl (coded 0) or boy (coded 1) for gender; month and year of birth (used to calculate age at the date of data collection); and parents’ birth countries. We measured migration background by distinguishing between adolescents with both parents born in the Netherlands (coded 0) and adolescents with at least one parent with a migration background (coded 1).

### Data Analyses

**Fig. 3:**
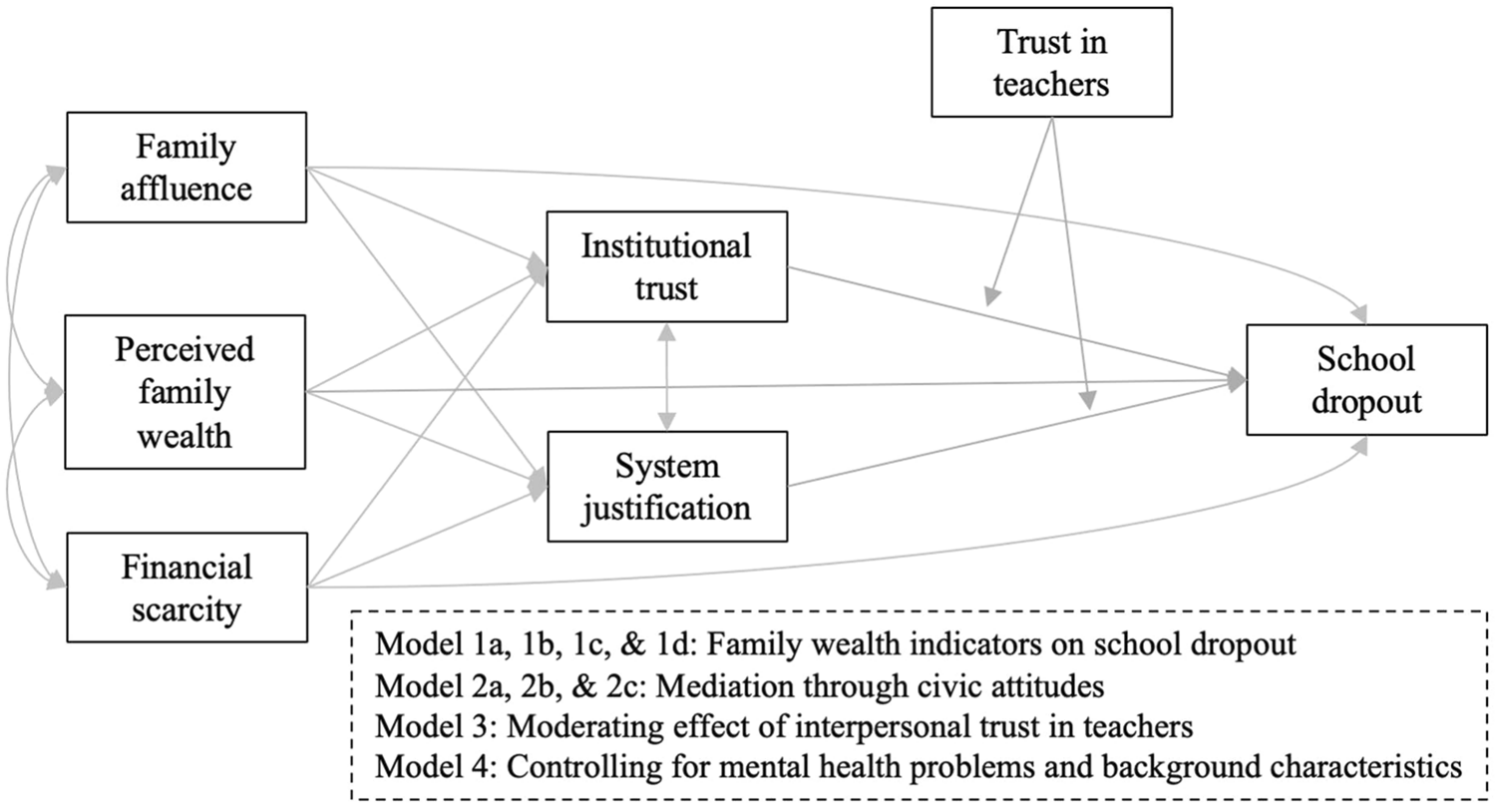
Hypothesized path model. The association between Family wealth indicators (i.e., family affluence, perceived family wealth, financial scarcity) and school dropout mediated by civic attitudes (i.e., system justification and institutional trust).

Hypotheses were tested with path analysis using Mplus 8.8^82^. Using a stepwise approach, we first entered *family SES*, *perceived family wealth*, and *financial scarcity* as continuous predictors of school dropout (1/0) in three separate models using logistic regression (Models 1a, 1b, 1c). Next, to test the independent associations between the three family wealth indicators and school dropout, we estimated their associations with school dropout simultaneously, including correlations among them (Model 1d). Subsequently, Model 1d was expanded with the civic attitudes *institutional trust* (Model 2a), *system justification* (Model 2b), and both simultaneously (Model 2c), as continuous mediators of the pathways between the family wealth indicators and school dropout. To examine whether interpersonal trust in teachers buffers the harmful effects of lower civic attitudes on school dropout, we expanded Model 2c with *trust in teachers* as a continuous moderator (Model 3). To rule out confounding, we expanded Model 3 with gender, age, immigrant background, and/or mental health problems (i.e., emotional, peer, and conduct problems and hyperactivity/inattention), depending on whether they were significantly correlated with school dropout (Model 4). Fig. 3 displays our hypothetical model.

Table 1 shows that out of 1,231 adolescents, information on school dropout was missing for 172 adolescents (13.97%), either because students did not provide permission to share this data (*n* = 146) or because the school did not provide the information (*n* = 26). The strength of the associations between the study variables and missing on dropout was (very) small (max. *r* = 0.09). Missing rates for the other study variables were (very) low, ranging from 0.08% (gender) to 7.07% (system justification). All models were estimated with Maximum Likelihood with robust standard errors (MLR) to non-normal distributions of the path model outcomes. This estimation method uses a full information approach and therefore retains all 1,231 adolescents in the models with all covariates included (M3,4a-c).

The data had a hierarchical structure, where individuals were nested in school classes (*n*_classes_ = 71*)*. Calculation of the intraclass correlations (ICC) showed substantial variance of some study variables at the school class level relative to the individual level (Table 1). Therefore, standard errors of the model estimates were corrected for school class clustering. To accommodate estimation of the path model with MLR, we used Monte Carlo integration with 5000 integration points^82^. Indirect effects were estimated using the product of relevant path coefficients, and total effects using the sum of the direct and indirect effects^66^. Significance of the indirect and total effects was evaluated using bootstrap 95% confidence intervals with 5000 replications^83^. Significance of the direct effects was evaluated using a *p-*value of 0.05. Associations were interpreted based on correlations and standardized coefficients (0.1 = small, 0.3 = medium, 0.5 = large effect^84^ and odds ratios transformed into probabilities. Model fit of the models were compared using Chi-square difference tests^85^ and changes in the Akaike Information Criterion (AIC) and Bayesian Information Criterion (BIC). Additional model specifications were applied to accommodate accurate model comparisons given our models (see Supplementary Table 3).

All models were estimated using manifest variables. The structural validity of the multiple-item measures was satisfactory. More information can be found in Supplementary Table 4. In addition, the analysis plan was preregistered. Deviations from the preregistration were applied to improve the analysis (see Supplementary Table 3). The preregistration and scripts of all analyses can be found at osf.io/ezjuf.

### Reporting summary

Further information on research design is available in the Nature Research Reporting Summary linked to this article.

### Supplementary information

Supplementary Tables

Reporting Summary

## Data Availability

The data supporting the findings of this study are available from the corresponding author CF upon a reasonable data request (i.e., the request does not violate the privacy protection of human subjects).
