# Supplementary material for: Examining the role of civic attitudes in the link between family wealth and school dropout among tertiary vocational students
Source: NPJ Sci Learn. 2023 Sep 15;8:38. doi: 10.1038/s41539-023-00189-4 (PMC10504291; doi:10.1038/s41539-023-00189-4)
Supplement: Supplementary file 1 — Supplementary Tables [file 41539_2023_189_MOESM1_ESM.pdf]

# Supplementary Tables

**Supplementary Table 1 Model fit indices**

| | Model fit | | | | | Model comparison | | | | | |
|---|---|---|---|---|---|---|---|---|---|---|---|
| | **p[a]** | **L** | **c** | **AIC** | **BIC** | **Compared to** | **cd[b]** | **df** | **Scaled Chi-square difference test[c]** | **ΔAIC** | **ΔBIC** |
| M1a | 24 | -20001.645 | 1.483 | 40051.290 | 40174.064 | | | | | | |
| M1b | 24 | -20003.037 | 1.486 | 40054.074 | 40176.848 | | | | | | |
| M1c | 24 | -20000.805 | 1.486 | 40049.609 | 40172.383 | | | | | | |
| M1d | 29 | -19781.760 | 1.413 | 39621.520 | 39769.872 | | | | | | |
| M2a | 33 | -19734.590 | 1.371 | 39535.181 | 39703.995 | M1d | 1.067 | 4 | 88.441 | -86.339 | -65.877 |
| M2b | 33 | -19726.500 | 1.372 | 39519.001 | 39687.815 | M1d | 1.071 | 4 | 103.210 | -102.519 | -82.057 |
| M2c | 38 | -19521.531 | 1.351 | 39119.062 | 39313.454 | M1d | 1.149 | 9 | 452.843 | -502.458 | -456.418 |
| M3 | 41 | -19520.838 | 1.330 | 39123.676 | 39333.415 | M2c | 1.062 | 3 | 1.305 | 4.614 | 19.961 |
| M4a (conduct problems) | 45 | -19487.250 | 1.307 | 39064.500 | 39294.701 | M3 | 1.071 | 4 | 62.731 | -59.176 | -38.714 |
| M4b (emotional problems) | 45 | -19434.269 | 1.306 | 38958.537 | 39188.738 | M3 | 1.058 | 4 | 163.573 | -165.139 | -144.677 |
| M4c (hyperactivity/ inattention) | 45 | -19489.162 | 1.305 | 39068.324 | 39298.526 | M3 | 1.048 | 4 | 60.430 | -55.352 | -34.889 |

M = model; p = number of free parameters; L = loglikelihood; c = scaling correction factor for MLR; AIC = Akaike Information Criterion; BIC = Bayesian Information Criterion; cd = difference test scaling correction; df = degrees of freedom.
[a] The number of free parameters equals the parameters from the respective model as well as the variances and means of the covariates from Models 4a-c, which was necessary to accommodate accurate model fit comparisons. See Table S3 for details.
[b] Calculated using (p0 * c0 - p1*c1)/(p0 - p1), where 0 is the more restricted model (i.e., with less free parameters) and 1 the comparison model (i.e., with more free parameters).
[c] Calculated using -2*(L0 - L1)/cd, where 0 is the more restricted model (i.e., with less free parameters) and 1 the comparison model (i.e., with more free parameters).

**Supplementary Table 2 Results path models**

| | | | Institutional trust | | | | School dropout | | | |
|---|---|---|---|---|---|---|---|---|---|---|
| **Model[a]** | ***n*[b]** | **Estimates** | ***B*** | ***SE*** | ***p*** | ***β*** | ***B*** | ***SE*** | ***p/[95% CI]*** | ***OR*** |
| 2a | 1,217 | Direct effects | | | | | | | | |
| | | Family affluence | 0.371 | 0.196 | 0.058 | 0.055 | -0.642 | 0.399 | 0.108 | 0.526 |
| | | Perceived family wealth | **0.270** | **0.099** | **0.007** | **0.105** | 0.068 | 0.144 | 0.638 | 1.070 |
| | | Financial scarcity | **-0.419** | **0.089** | **<0.001** | **-0.173** | 0.211 | 0.137 | 0.122 | 1.235 |
| | | Indirect effects via institutional trust | | | | | | | | |
| | | Family affluence | | | | | -0.072 | 0.043 | [-0.166, 0.004] | 0.931 |
| | | Perceived family wealth | | | | | **-0.052** | **0.023** | **[-0.104,-0.015]** | **0.949** |
| | | Financial scarcity | | | | | **0.081** | **0.028** | **[0.032, 0.144]** | **1.084** |
| | | Total effects | | | | | | | | |
| | | Family affluence | | | | | -0.714 | 0.396 | [-1.498, 0.053] | 0.490 |
| | | Perceived family wealth | | | | | 0.015 | 0.150 | [-0.279, 0.309] | 1.016 |
| | | Financial scarcity | | | | | **0.292** | **0.135** | **[0.036, 0.559]** | **1.339** |
| | | Institutional trust | | | | | **-0.193** | **0.051** | **<0.001** | **0.824** |
| 2b | 1,218 | Direct effects | | | | | | | | |
| | | Family affluence | -0.031 | 0.104 | 0.769 | -0.009 | -0.708 | 0.399 | 0.076 | 0.493 |
| | | Perceived family wealth | **0.173** | **0.054** | **0.001** | **0.128** | 0.037 | 0.155 | 0.814 | 1.037 |
| | | Financial scarcity | **-0.288** | **0.039** | **<0.001** | **-0.228** | 0.239 | 0.145 | 0.099 | 1.270 |
| | | Indirect effects via system justification | | | | | | | | |
| | | Family affluence | | | | | 0.006 | 0.024 | [-0.039, 0.061] | 1.006 |
| | | Perceived family wealth | | | | | **-0.034** | **0.021** | **[-0.081, -0.001]** | **0.967** |
| | | Financial scarcity | | | | | **0.056** | **0.030** | **[0.002, 0.120]** | **1.058** |
| | | Total effects | | | | | | | | |
| | | Family affluence | | | | | -0.702 | 0.394 | [-1.478, 0.069] | 0.496 |
| | | Perceived family wealth | | | | | 0.003 | 0.156 | [-0.302, 0.314] | 1.003 |
| | | Financial scarcity | | | | | **0.295** | **0.136** | **[0.036, 0.560]** | **1.344** |
| | | System justification | | | | | **-0.195** | **0.098** | **0.046** | **0.823** |

*Note*s. Data were analyzed in MPlus 8.8 to perform path analyses using a stepwise approach, a specific subset of structural equation modeling (SEM). All models were estimated with Maximum Likelihood with robust standard errors (MLR) to non-normal distributions of the path model outcomes. SE = standard error; p = p-value; β = standardized coefficient; CI = confidence interval; OR = odds ratio. SEs and CIs are based on bootstrap with 5000 draws. Boldface rows denote significant paths.

[a] Because full information maximum likelihood was used, sample size *n* varied depending on the included variables.

[b] The model includes covariances between the three family wealth indicators (not shown in Table).

**Supplementary Table 2 (continued) Results path models**

| | | | **Institutional trust** | | | | **System justification** | | | | **School dropout** | | | |
|---|---|---|---|---|---|---|---|---|---|---|---|---|---|---|
| **Model[a]** | ***n*[b]** | **Estimates** | ***B*** | ***SE*** | ***p*** | ***β*** | ***B*** | ***SE*** | ***p*** | ***β*** | ***B*** | ***SE*** | ***p/[95% CI]*** | ***OR*** |
| 2c | 1,218 | Direct effects | | | | | | | | | | | | |
| | | Family affluence | 0.371 | 0.196 | 0.058 | 0.055 | -0.027 | 0.104 | 0.795 | -0.008 | -0.640 | 0.400 | 0.110 | 0.528 |
| | | Perceived family wealth | **0.276** | **0.100** | **0.006** | **0.107** | **0.178** | **0.054** | **0.001** | **0.132** | 0.070 | 0.144 | 0.628 | 1.072 |
| | | Financial scarcity | **-0.416** | **0.089** | **<0.001** | **-0.172** | **-0.285** | **0.038** | **<0.001** | **-0.226** | 0.205 | 0.142 | 0.149 | 1.228 |
| | | | | | | | | | | | | | | |
| | | Indirect effects via institutional trust | | | | | | | | | | | | |
| | | Family affluence | | | | | | | | | -0.073 | 0.049 | [-0.185, 0.004] | 0.929 |
| | | Perceived family wealth | | | | | | | | | **-0.055** | **0.026** | **[-0.113, -0.012]** | **0.947** |
| | | Financial scarcity | | | | | | | | | **0.083** | **0.034** | **[0.024, 0.156]** | **1.086** |
| | | | | | | | | | | | | | | |
| | | Indirect effects via system justification | | | | | | | | | | | | |
| | | Family affluence | | | | | | | | | 0.000 | 0.013 | [-0.026, 0.030] | 1.000 |
| | | Perceived family wealth | | | | | | | | | -0.001 | 0.023 | [-0.050, 0.041] | 0.999 |
| | | Financial scarcity | | | | | | | | | 0.002 | 0.035 | [-0.064, 0.074] | 1.002 |
| | | | | | | | | | | | | | | |
| | | Total effects | | | | | | | | | | | | |
| | | Family affluence | | | | | | | | | -0.713 | 0.397 | [-1.499, 0.060] | 0.490 |
| | | Perceived family wealth | | | | | | | | | 0.014 | 0.150 | [-0.281, 0.310] | 1.014 |
| | | Financial scarcity | | | | | | | | | **0.289** | **0.135** | **[0.034, 0.555]** | **1.335** |
| | | | | | | | | | | | | | | |
| | | Institutional trust | | | | | | | | | **-0.198** | **0.065** | **0.002** | **0.820** |
| | | System justification | | | | | | | | | -0.005 | 0.123 | 0.965 | 0.995 |

*Note*s. Data were analyzed in MPlus 8.8 to perform path analyses using a stepwise approach, a specific subset of structural equation modeling (SEM). All models were estimated with Maximum Likelihood with robust standard errors (MLR) to non-normal distributions of the path model outcomes. SE = standard error; p = p-value; β = standardized coefficient; CI = confidence interval; OR = odds ratio. SEs and CIs are based on bootstrap with 5000 draws. Boldface rows denote significant paths.

[a] Because full information maximum likelihood was used, sample size *n* varied depending on the included variables.

[b] The model includes covariances between the three family wealth indicators and covariances between the two civic attitudes (not shown in Table).

**Supplementary Table 2 (continued) Results path models**

| | | | **Institutional trust** | | | | **System justification** | | | | **School dropout** | | | |
|---|---|---|---|---|---|---|---|---|---|---|---|---|---|---|
| **Model[a]** | ***n*[b]** | **Estimates** | ***B*** | ***SE*** | ***p*** | ***β*** | ***B*** | ***SE*** | ***p*** | ***β*** | ***B*** | ***SE*** | ***p/[95% CI]*** | ***OR*** |
| 3 | 1,231 | Direct effects | | | | | | | | | | | | |
| | | Family affluence | 0.370 | 0.196 | 0.058 | 0.055 | -0.026 | 0.104 | 0.800 | -0.008 | -0.686 | 0.418 | 0.100 | 0.503 |
| | | Perceived family wealth | **0.276** | **0.100** | **0.006** | **0.107** | **0.178** | **0.054** | **0.001** | **0.132** | 0.058 | 0.148 | 0.696 | 1.059 |
| | | Financial scarcity | **-0.417** | **0.089** | **<0.001** | **-0.172** | **-0.285** | **0.038** | **<0.001** | **-0.226** | 0.208 | 0.143 | 0.147 | 1.231 |
| | | | | | | | | | | | | | | |
| | | Indirect effects via institutional trust | | | | | | | | | | | | |
| | | Family affluence | | | | | | | | | -0.065 | 0.047 | [-0.176, 0.005] | 0.937 |
| | | Perceived family wealth | | | | | | | | | **-0.049** | **0.026** | **[-0.108, -0.008]** | **0.952** |
| | | Financial scarcity | | | | | | | | | **0.074** | **0.034** | **[0.016, 0.148]** | **1.076** |
| | | | | | | | | | | | | | | |
| | | Indirect effects via system justification | | | | | | | | | | | | |
| | | Family affluence | | | | | | | | | 0.000 | 0.014 | [-0.028, 0.031] | 1.000 |
| | | Perceived family wealth | | | | | | | | | 0.001 | 0.024 | [-0.049, 0.048] | 1.001 |
| | | Financial scarcity | | | | | | | | | -0.002 | 0.037 | [-0.077, 0.071] | 0.998 |
| | | | | | | | | | | | | | | |
| | | Total effects | | | | | | | | | | | | |
| | | Family affluence | | | | | | | | | -0.752 | 0.415 | [-1.584, 0.034] | 0.471 |
| | | Perceived family wealth | | | | | | | | | 0.010 | 0.152 | [-0.288, 0.307] | 1.010 |
| | | Financial scarcity | | | | | | | | | **0.280** | **0.135** | **[0.023, 0.542]** | **1.323** |
| | | | | | | | | | | | | | | |
| | | Institutional trust | | | | | | | | | **-0.176** | **0.069** | **0.011** | **0.838** |
| | | System justification | | | | | | | | | 0.005 | 0.130 | 0.966 | 1.005 |
| | | | | | | | | | | | | | | |
| | | Trust in teachers | | | | | | | | | -0.121 | 0.137 | 0.377 | 0.886 |
| | | Institutional trust * Trust in teachers | | | | | | | | | 0.044 | 0.085 | 0.605 | 1.045 |
| | | System justification * Trust in teachers | | | | | | | | | 0.003 | 0.177 | 0.985 | 1.003 |

*Note*s. Data were analyzed in MPlus 8.8 to perform path analyses using a stepwise approach, a specific subset of structural equation modeling (SEM). All models were estimated with Maximum Likelihood with robust standard errors (MLR) to non-normal distributions of the path model outcomes. SE = standard error; p = p-value; β = standardized coefficient; CI = confidence interval; OR = odds ratio. SEs and CIs are based on bootstrap with 5000 draws. Boldface rows denote significant paths.

[a] Because full information maximum likelihood was used, sample size *n* varied depending on the included variables.

[b] The model includes covariances between the three family wealth indicators and covariances between the two civic attitudes (not shown in Table).

**Supplementary Table 2 (continued) Results path models**

| | | | Institutional trust | | | | System justification | | | | School dropout | | | |
|---|---|---|---|---|---|---|---|---|---|---|---|---|---|---|
| **Model[a]** | ***n*[b]** | **Estimates** | ***B*** | ***SE*** | ***p*** | ***β*** | ***B*** | ***SE*** | ***p*** | ***β*** | ***B*** | ***SE*** | ***p/[95% CI]*** | ***OR*** |
| 4a | 1,231 | Direct effects | | | | | | | | | | | | |
| | | Family affluence | 0.372 | 0.196 | 0.057 | 0.056 | -0.026 | 0.104 | 0.801 | -0.007 | -0.731 | 0.423 | 0.084 | 0.481 |
| | | Perceived family wealth | **0.276** | **0.100** | **0.006** | **0.107** | **0.177** | **0.054** | **0.001** | **0.132** | 0.047 | 0.148 | 0.750 | 1.049 |
| | | Financial scarcity | **-0.417** | **0.089** | **<0.001** | **-0.172** | **-0.285** | **0.038** | **<0.001** | **-0.226** | 0.157 | 0.144 | 0.277 | 1.170 |
| | | Indirect effects via institutional trust | | | | | | | | | | | | |
| | | Family affluence | | | | | | | | | -0.062 | 0.046 | [-0.169, 0.006] | |
| | | Perceived family wealth | | | | | | | | | **-0.046** | **0.025** | **[-0.103, -0.006]** | |
| | | Financial scarcity | | | | | | | | | **0.069** | **0.034** | **[0.011, 0.145]** | |
| | | Indirect effects via system justification | | | | | | | | | | | | |
| | | Family affluence | | | | | | | | | 0.000 | 0.014 | [-0.029, 0.032] | |
| | | Perceived family wealth | | | | | | | | | 0.002 | 0.024 | [-0.048, 0.049] | |
| | | Financial scarcity | | | | | | | | | -0.004 | 0.038 | [-0.080, 0.070] | |
| | | Total effects | | | | | | | | | | | | |
| | | Family affluence | | | | | | | | | -0.794 | 0.420 | [-1.634, 0.007] | |
| | | Perceived family wealth | | | | | | | | | 0.004 | 0.150 | [-0.291, 0.294] | |
| | | Financial scarcity | | | | | | | | | 0.223 | 0.140 | [-0.048, 0.496] | |
| | | Institutional trust | | | | | | | | | **-0.167** | **0.070** | **0.018** | **0.847** |
| | | System justification | | | | | | | | | 0.013 | 0.132 | 0.922 | 1.013 |
| | | Trust in teachers | | | | | | | | | -0.088 | 0.143 | 0.536 | 0.916 |
| | | Institutional trust * Trust in teachers | | | | | | | | | 0.044 | 0.086 | 0.605 | 1.045 |
| | | System justification * Trust in teachers | | | | | | | | | -0.006 | 0.180 | 0.974 | 0.994 |
| | | Conduct problems | | | | | | | | | 0.134 | 0.077 | 0.082 | 1.144 |

*Note*s. Data were analyzed in MPlus 8.8 to perform path analyses using a stepwise approach, a specific subset of structural equation modeling (SEM). All models were estimated with Maximum Likelihood with robust standard errors (MLR) to non-normal distributions of the path model outcomes. SE = standard error; p = p-value; β = standardized coefficient; CI = confidence interval; OR = odds ratio. SEs and CIs are based on bootstrap with 5000 draws. Boldface rows denote significant paths.

[a] Because full information maximum likelihood was used, sample size *n* varied depending on the included variables.

[b] The model includes covariances between the three family wealth indicators and conduct problems, and covariances between the two civic attitudes (not shown in Table).

**Supplementary Table 2 (continued) Results path models**

| | | | Institutional trust | | | | System justification | | | | School dropout | | | |
|---|---|---|---|---|---|---|---|---|---|---|---|---|---|---|
| **Model[a]** | ***n*[b]** | **Estimates** | ***B*** | ***SE*** | ***p*** | ***β*** | ***B*** | ***SE*** | ***p*** | ***β*** | ***B*** | ***SE*** | ***p/[95% CI]*** | ***OR*** |
| 4b | 1,231 | Direct effects | | | | | | | | | | | | |
| | | Family affluence | 0.372 | 0.196 | 0.057 | 0.056 | -0.027 | 0.104 | 0.797 | -0.008 | -0.660 | 0.417 | 0.113 | 0.517 |
| | | Perceived family wealth | **0.277** | **0.100** | **0.006** | **0.107** | **0.177** | **0.054** | **0.001** | **0.131** | 0.048 | 0.150 | 0.748 | 1.049 |
| | | Financial scarcity | **-0.416** | **0.089** | **<0.001** | **-0.172** | **-0.285** | **0.038** | **<0.001** | **-0.226** | 0.149 | 0.147 | 0.310 | 1.161 |
| | | | | | | | | | | | | | | |
| | | Indirect effects via institutional trust | | | | | | | | | | | | |
| | | Family affluence | | | | | | | | | -0.065 | 0.047 | [-0.175, 0.005] | |
| | | Perceived family wealth | | | | | | | | | **-0.049** | **0.026** | **[-0.108, -0.007]** | |
| | | Financial scarcity | | | | | | | | | **0.073** | **0.034** | **[0.014, 0.149]** | |
| | | | | | | | | | | | | | | |
| | | Indirect effects via system justification | | | | | | | | | | | | |
| | | Family affluence | | | | | | | | | -0.001 | 0.014 | [-0.031, 0.032] | |
| | | Perceived family wealth | | | | | | | | | 0.004 | 0.025 | [-0.045, 0.054] | |
| | | Financial scarcity | | | | | | | | | -0.007 | 0.038 | [-0.084, 0.066] | |
| | | | | | | | | | | | | | | |
| | | Total effects | | | | | | | | | | | | |
| | | Family affluence | | | | | | | | | -0.726 | 0.414 | [-1.560, 0.057] | |
| | | Perceived family wealth | | | | | | | | | 0.004 | 0.153 | [-0.293, 0.304] | |
| | | Financial scarcity | | | | | | | | | 0.215 | 0.141 | [-0.060, 0.491] | |
| | | | | | | | | | | | | | | |
| | | Institutional trust | | | | | | | | | **-0.175** | **0.070** | **0.013** | **0.839** |
| | | System justification | | | | | | | | | 0.025 | 0.133 | 0.849 | 1.026 |
| | | | | | | | | | | | | | | |
| | | Trust in teachers | | | | | | | | | -0.101 | 0.139 | 0.468 | 0.904 |
| | | Institutional trust * Trust in teachers | | | | | | | | | 0.036 | 0.085 | 0.668 | 1.037 |
| | | System justification * Trust in teachers | | | | | | | | | 0.011 | 0.176 | 0.950 | 1.011 |
| | | | | | | | | | | | | | | |
| | | Emotional problems | | | | | | | | | 0.054 | 0.038 | 0.156 | 1.056 |

*Note*s. Data were analyzed in MPlus 8.8 to perform path analyses using a stepwise approach, a specific subset of structural equation modeling (SEM). All models were estimated with Maximum Likelihood with robust standard errors (MLR) to non-normal distributions of the path model outcomes. SE = standard error; p = p-value; β = standardized coefficient; CI = confidence interval; OR = odds ratio. SEs and CIs are based on bootstrap with 5000 draws. Boldface rows denote significant paths.

[a] Because full information maximum likelihood was used, sample size *n* varied depending on the included variables.

[b] The model includes covariances between the three family wealth indicators and emotional problems, and covariances between the two civic attitudes (not shown in Table).

**Supplementary Table 2 (continued) Results path models**

| | | | Institutional trust | | | | System justification | | | | School dropout | | | |
|---|---|---|---|---|---|---|---|---|---|---|---|---|---|---|
| **Model[a]** | ***n*[b]** | **Estimates** | ***B*** | ***SE*** | ***p*** | ***β*** | ***B*** | ***SE*** | ***p*** | ***β*** | ***B*** | ***SE*** | ***p/[95% CI]*** | ***OR*** |
| 4c | 1,231 | Direct effects | | | | | | | | | | | | |
| | | Family affluence | 0.373 | 0.196 | 0.057 | 0.056 | -0.025 | 0.104 | 0.813 | -0.007 | -0.803 | 0.421 | 0.056 | 0.448 |
| | | Subjective family wealth | **0.277** | **0.099** | **0.005** | **0.107** | **0.177** | **0.054** | **0.001** | **0.131** | 0.049 | 0.150 | 0.743 | 1.050 |
| | | Financial scarcity | **-0.416** | **0.089** | **<0.001** | **-0.172** | **-0.285** | **0.038** | **<0.001** | **-0.226** | 0.150 | 0.144 | 0.297 | 1.162 |
| | | | | | | | | | | | | | | |
| | | Indirect effects via institutional trust | | | | | | | | | | | | |
| | | Family affluence | | | | | | | | | -0.064 | 0.046 | [-0.170, 0.005] | |
| | | Perceived family wealth | | | | | | | | | **-0.048** | **0.025** | **[-0.104, -0.007]** | |
| | | Financial scarcity | | | | | | | | | **0.072** | **0.034** | **[0.014, 0.147]** | |
| | | | | | | | | | | | | | | |
| | | Indirect effects via system justification | | | | | | | | | | | | |
| | | Family affluence | | | | | | | | | 0.000 | 0.014 | [-0.030, 0.032] | |
| | | Perceived family wealth | | | | | | | | | 0.002 | 0.024 | [-0.048, 0.051] | |
| | | Financial scarcity | | | | | | | | | -0.004 | 0.038 | [-0.081, 0.070] | |
| | | | | | | | | | | | | | | |
| | | Total effects | | | | | | | | | | | | |
| | | Family affluence | | | | | | | | | **-0.867** | **0.419** | **[-1.721, -0.079]** | |
| | | Perceived family wealth | | | | | | | | | 0.004 | 0.153 | [-0.295, 0.306] | |
| | | Financial scarcity | | | | | | | | | 0.218 | 0.137 | [-0.044, 0.483] | |
| | | | | | | | | | | | | | | |
| | | Institutional trust | | | | | | | | | -0.172 | 0.069 | 0.013 | 0.842 |
| | | System justification | | | | | | | | | 0.014 | 0.133 | 0.919 | 1.014 |
| | | | | | | | | | | | | | | |
| | | Trust in teachers | | | | | | | | | -0.088 | 0.136 | 0.520 | 0.916 |
| | | Institutional trust * Trust in teachers | | | | | | | | | 0.039 | 0.086 | 0.650 | 1.040 |
| | | System justification * Trust in teachers | | | | | | | | | 0.005 | 0.181 | 0.977 | 1.005 |
| | | | | | | | | | | | | | | |
| | | Hyperactivity/inattention | | | | | | | | | 0.082 | 0.036 | 0.020 | 1.086 |

*Notes*. Data were analyzed in MPlus 8.8 to perform path analyses using a stepwise approach, a specific subset of structural equation modeling (SEM). All models were estimated with Maximum Likelihood with robust standard errors (MLR) to non-normal distributions of the path model outcomes. SE = standard error; p = p-value; β = standardized coefficient; CI = confidence interval; OR = odds ratio. SEs and CIs are based on bootstrap with 5000 draws. Boldface rows denote significant paths.

[a] Because full information maximum likelihood was used, sample size *n* varied depending on the included variables.

[b] The model includes covariances between the three family wealth indicators and hyperactivity/inattention, and covariances between the two civic attitudes (not shown in Table).

**Supplementary Table 3 Deviations from preregistration**

| What was modified | Plan according preregistration | Modification | Reason for modification |
|---|---|---|---|
| Testing confounding | Test confounding of migration background, emotional problems, peer problems, conduct problems, and hyperactivity/inattention | We only tested confounding of these variables when they were significantly correlated with school dropout. We additionally tested this for gender and age. | Test less models |
| Sample size | Using the entire sample of students who participated in Wave 1 and whom we received the dropout information (*n* = 1,059). | Cases with missing data on dropout were additionally included. That is, those who did not give the teachers permission to share their dropout data with the researchers and cases for which information on school dropout was not received, leading to a total sample of 1,231. | Limit bias by retaining more data. |
| Calculation of SEs | Not specified | Given the hierarchical nature of the data, where respondents are nested within school classes (n = 71), a correction for school clustering was applied on the standard errors of the model estimates. | Obtain more reliable SEs. |
| Integration specification | Not specified | Model estimations were conducted using Monte Carlo integration with 5000 integration points. | Testing our path model with MLR estimation with missing data, categorical and continuous outcomes required Monte Carlo integration. |

The preregistration can be found at osf.io/ezjuf

**Supplementary Table 3 (continued) Deviations from preregistration**

| | | | |
|---|---|---|---|
| Significance test | Use p-values < 0.05 to evaluate hypotheses | Model estimates were conducted using bootstrap with 5000 replications. Significance of the indirect and total effects were evaluated based on the 95% bootstrap confidence intervals instead of p-values. | Test significance of non-normally distributed effects accurately in line with recommendations for mediation analyses. Indirect and total effects are not normally distributed because they include the product of relevant paths. |
| Interpretation of the effects on school dropout | Transform logit coefficients into odds ratios. | Add interpretation in terms of probabilities. | Facilitate interpretability of the results: providing insight into predicted probabilities of dropout given certain values of family wealth indicators, which may be more informative than differences in relative risk (as with odds ratios). |
| Model fit evaluation | Evaluate using Chi-square difference test, AIC, and BIC | These indices were used, but additional model specifications were applied to accommodate model fit comparisons, which were not preregistered. Specifically, we estimated Models 1a-d until 3 with all variables from Models 4a-c, but with all paths constrained to 0 except the hypothesized paths from the respective model. Model fit indices were obtained from these models. | Facilitate accurate model fit comparison. Chi-square difference test require nested models. To be nested, models should have the same set of observed variables, which was not the case in our models. AIC and BIC can be used to compare non-nested models, but only when the outcomes are the same, which was also not the case for out models. The additional specification transforms the models into nested models, that share all outcomes. |

The preregistration can be found at osf.io/ezjuf

**Supplementary Table 4 CFA multiple-item measures**

| Family affluence | Item | Factor loading | *SE* | *p* |
|---|---|---|---|---|
| Model fit:<br>CFI = 1.000; TLI = 1.000; RMSEA = < 0.001; SRMR = 0.021 | Does your family own a car (or a (van))? | 0.641 | 0.040 | < 0.001 |
| | Do you have your own bedroom (for you alone)? | 0.428 | 0.069 | < 0.001 |
| | How many computers does your family have (laptops, iPads and tablets do count, smartphones, game consoles do not) | 0.645 | 0.043 | < 0.001 |
| | How many bathrooms (with a shower or bathtub) does your house have? | 0.579 | 0.047 | < 0.001 |
| | Do you have a dishwasher at home? | 0.658 | 0.049 | < 0.001 |
| | In the last 12 months, how often did you and your family go on vacation outside the Netherlands (a short or long vacation)? | 0.319 | 0.042 | < 0.001 |
| **Financial scarcity** | **Item** | **Factor loading** | ***SE*** | ***p*** |
| Model fit:<br>CFI = 0.948; TLI = 0.914; RMSEA = 0.088; SRMR = 0.036 | I am often short of money. | 0.730 | 0.018 | < 0.001 |
| | I wonder all the time if I have enough money. | 0.831 | 0.020 | < 0.001 |
| | I often worry about money. | 0.763 | 0.024 | < 0.001 |
| | I'm only concerned with what I have to pay now. The rest I'll see later. | 0.327 | 0.033 | < 0.001 |
| | I feel I have little control over my money matters. | 0.500 | 0.026 | < 0.001 |
| | In my family, there are often worries about money. | 0.522 | 0.031 | < 0.001 |

*Notes*. Confirmatory factor analyses for all measures with more than 3 items using MPlus 8.8. Model fit of three-item factors could not be assessed because these models were just-identified, i.e., had no degrees of freedom. Acceptable model fit was indicated by CFI/TLI >= 0.900, RMSEA <= 0.080, SRMR <= 0.100 (Hu & Bentler, 1999).
[a] Item was reverse coded prior to scale construction.
*SE* = standard error; p = *p*-value; CFI = Comparative Fit Index, TLI = Tucker Lewis Index; RMSEA = Root Mean Square Error of Approximation; SRMR = Standardized Root Mean Square Residual.

**Supplementary Table 4 (continued) CFA multiple-item measures**

| System justification | Item | Factor loading | *SE* | *p* |
|---|---|---|---|---|
| Model fit:<br>CFI = 0.925; TLI = 0.906; RMSEA = 0.078; SRMR = 0.039 | In general, Dutch society is fair. | 0.678 | 0.020 | < 0.001 |
| | The Netherlands is a country where anyone who works hard can get ahead. | 0.677 | 0.022 | < 0.001 |
| | Mostly, the Dutch government works as it should work. | 0.741 | 0.019 | < 0.001 |
| | In the Netherlands, you have equal opportunities no matter where you come from and who you are. | 0.728 | 0.019 | < 0.001 |
| | The Netherlands is the best country to live in. | 0.652 | 0.024 | < 0.001 |
| | Most laws and policies in the Netherlands are good for most people. | 0.753 | 0.020 | < 0.001 |
| | In the Netherlands, everyone has a fair chance to get enough money. | 0.791 | 0.016 | < 0.001 |
| | In the Netherlands, everyone has a fair chance to be happy. | 0.751 | 0.023 | < 0.001 |
| | In the Netherlands, society is arranged in such a way that people usually get what they are entitled to. | 0.820 | 0.015 | < 0.001 |
| | People are treated fairly in the Netherlands no matter who they are. | 0.741 | 0.021 | < 0.001 |
| | It is not right that in the Netherlands there are very rich and very poor people.[a] | 0.081 | 0.040 | 0.042 |
| **Institutional trust** | **Item** | **Factor loading** | ***SE*** | ***p*** |
| Model fit:<br>CFI = 0.936; TLI = 904; RMSEA = 0.097; SRMR = 0.040 | I trust: Dutch politicians | 0.804 | 0.014 | < 0.001 |
| | I trust: The police | 0.785 | 0.017 | < 0.001 |
| | I trust: Caregivers, e.g., doctors, psychologists, etc. | 0.602 | 0.040 | < 0.001 |
| | I trust: People working in government (civil servants) | 0.875 | 0.012 | < 0.001 |
| | I trust: The News | 0.738 | 0.018 | < 0.001 |
| | I trust: Courts and judges | 0.791 | 0.018 | < 0.001 |
| | I trust: Information on social media | 0.558 | 0.025 | < 0.001 |

*Notes*. Confirmatory factor analyses for all measures with more than 3 items using MPlus 8.8. Model fit of three-item factors could not be assessed because these models were just-identified, i.e., had no degrees of freedom. Acceptable model fit was indicated by CFI/TLI >= 0.900, RMSEA <= 0.080, SRMR <= 0.100 (Hu & Bentler, 1999).
[a] Item was reverse coded prior to scale construction.
*SE* = standard error; p = *p*-value; CFI = Comparative Fit Index, TLI = Tucker Lewis Index; RMSEA = Root Mean Square Error of Approximation; SRMR = Standardized Root Mean Square Residual.

**Supplementary Table 4 (continued) CFA multiple-item measures**

| **Interpersonal trust in teachers** | **Item** | **Factor loading** | ***SE*** | ***p*** |
|---|---|---|---|---|
| | I feel that my teachers accept me as I am. | 0.695 | 0.027 | < 0.001 |
| | I feel that my teachers care about me. | 0.822 | 0.018 | < 0.001 |
| | I trust my teachers. | 0.842 | 0.018 | < 0.001 |
| **Emotional symptoms** | **Item** | **Factor loading** | ***SE*** | ***p*** |
| Model fit:<br>CFI = 0.986; TLI = 0.971; RMSEA = 0.073; SRMR = 0.028 | I often have a headache, stomachache, or am nauseous. | 0.534 | 0.027 | < 0.001 |
| | I worry a lot | 0.700 | 0.022 | < 0.001 |
| | I am often unhappy, depressed or in tears. | 0.752 | 0.030 | < 0.001 |
| | I am nervous in new situations. I lose confidence easily. | 0.719 | 0.026 | < 0.001 |
| | I am afraid of many things, I am easily anxious. | 0.795 | 0.025 | < 0.001 |
| **Conduct problems** | **Item** | **Factor loading** | ***SE*** | ***p*** |
| Model fit:<br>CFI = 1.000; TLI = 1.000; RMSEA = < 0.001; SRMR = 0.017 | I get very angry and often have a temper. | 0.551 | 0.070 | < 0.001 |
| | I often fight. I manage to get other people to do what I want. | 0.565 | 0.069 | < 0.001 |
| | I am often accused of lying or cheating. | 0.585 | 0.070 | < 0.001 |
| **Hyperactivity/inattention** | **Item** | **Factor loading** | ***SE*** | ***p*** |
| | I am restless, I can't sit still for a long time. | 0.805 | 0.024 | < 0.001 |
| | I am constantly wiggling or fidgeting. | 0.797 | 0.027 | < 0.001 |
| | I am easily distracted, I find it difficult to concentrate. | 0.654 | 0.028 | < 0.001 |
| **Peer relationship problems** | **Item** | **Factor loading** | ***SE*** | ***p*** |
| | I'm pretty much on my own. I usually play alone or don't interfere with others. | 0.471 | 0.077 | < 0.001 |
| | Other children or young people bully or harass me. | 0.607 | 0.079 | < 0.001 |
| | I get along better with adults than with young people my age. | 0.489 | 0.083 | < 0.001 |

*Notes*. Confirmatory factor analyses for all measures with more than 3 items using MPlus 8.8. Model fit of three-item factors could not be assessed because these models were just-identified, i.e., had no degrees of freedom. Acceptable model fit was indicated by CFI/TLI >= 0.900, RMSEA <= 0.080, SRMR <= 0.100 (Hu & Bentler, 1999).
[a] Item was reverse coded prior to scale construction.
*SE* = standard error; p = *p*-value; CFI = Comparative Fit Index, TLI = Tucker Lewis Index; RMSEA = Root Mean Square Error of Approximation; SRMR = Standardized Root Mean Square Residual.
